# PCSK9 at the intersection of lipid metabolism, immunometabolism, and psychiatric disorders: A narrative review

**DOI:** 10.1016/j.bbih.2026.101289

**Published:** 2026-06-20

**Authors:** Folkert H. van Bruggen, Roger S. McIntyre

**Affiliations:** a Department of Primary and Long-Term Care, University Medical Centre Groningen, University of Groningen, P.O. Box 196, Groningen, 9700 AD, the Netherlands; b Department of Psychiatry, University of Toronto, Ontario, Canada; c Department of Pharmacology and Toxicology, University of Toronto, Toronto, Ontario, Canada

**Keywords:** PCSK9, Immunometabolism, Dyslipidemia, Neuroinflammation, Psychiatric disorders, Cholesterol homeostasis

## Abstract

Proprotein convertase subtilisin/kexin type 9 (PCSK9) is a central regulator of lipid metabolism and a well-established determinant of cardiovascular risk. In addition to its hepatic role in cholesterol homeostasis, PCSK9 is increasingly linked to immunometabolic processes that overlap with biological alterations commonly observed in psychiatric disorders, including dyslipidemia, insulin resistance, and low-grade systemic and neuroinflammation.

This narrative review synthesizes experimental, genetic, and clinical evidence on lipid metabolism and PCSK9-related pathways in psychiatric disorders. Disorder-specific patterns of peripheral lipid abnormalities are reviewed alongside evidence for altered central lipid composition and cholesterol turnover in major psychiatric and neuropsychiatric conditions. Available data on PCSK9 expression and regulation within the central nervous system (CNS) are summarized, together with studies examining circulating PCSK9 levels in relation to metabolic burden, inflammation, and psychiatric symptomatology.

Overall, the literature positions PCSK9 within interconnected metabolic and inflammatory pathways associated with psychiatric vulnerability, primarily as a peripheral marker. Although PCSK9 is implicated in neuroinflammation and may intersect with pathways relevant to brain insulin resistance, its role within the CNS remains incompletely characterized in relation to psychiatric disorders, highlighting an important area for future mechanistic and translational research.

## Highlights

• Peripheral PCSK9 emerges primarily as a marker of immunometabolic burden in psychiatric disorders.
• Although PCSK9 has been implicated in central nervous system processes such as cholesterol regulation and inflammatory signaling, its role in human psychiatric disorders remains poorly defined.
• PCSK9 provides a framework linking dyslipidemia, neuroinflammation, and metabolic dysfunction in psychiatry.

## 1 Introduction

Second-generation antipsychotics (SGAs) are widely used in the treatment of schizophrenia and related psychiatric disorders but are frequently associated with adverse metabolic effects, including weight gain, insulin resistance, and dyslipidemia (Pillinger et al., 2020). Among these agents, olanzapine is particularly well known for its pronounced impact on lipid metabolism. Recent evidence indicates that olanzapine-associated dyslipidemia is accompanied by increased circulating levels of proprotein convertase subtilisin/kexin type 9 (PCSK9), a key regulator of low-density lipoprotein receptor (LDLR) turnover and plasma LDL-cholesterol (LDL-C) concentrations (J. Huang et al., 2022; P. Huang et al., 2024; Zhu et al., 2022). Although identified in the context of antipsychotic-associated dysmetabolism, this observation raises the possibility that PCSK9-related pathways may have broader relevance to immunometabolic and neurobiological processes implicated across psychiatric disorders. At present, the available evidence remains dispersed across disciplines and has not yet been critically examined within an integrative framework.

PCSK9 has traditionally been studied in the context of cardiovascular disease, where its role in hepatic LDLR degradation and lipid homeostasis is well established (Seidah and Prat, 2012). However, emerging evidence suggests that PCSK9 may also be relevant beyond cardiometabolic risk, owing to its involvement in systemic inflammation, cellular cholesterol trafficking, and extra-hepatic tissues, including the CNS. Although PCSK9 expression in the brain is low under physiological conditions, experimental studies indicate that it may influence neuronal lipid handling (Pärn et al., 2023), neuroinflammatory signaling (Vilella et al., 2024), and receptor systems implicated in synaptic function (Pelucchi et al., 2025). These observations raise the possibility that PCSK9-related pathways could intersect with biological processes increasingly recognized as relevant to psychiatric disorders.

In parallel, a growing literature has documented dysregulated lipid metabolism across a range of psychiatric conditions, including major depressive disorder (MDD) (Bharti et al., 2021; Persons and Fiedorowicz, 2016; Xu et al., 2024), psychotic disorders (Pillinger et al., 2017; Wang et al., 2024), and post-traumatic stress disorder (Bharti et al., 2022). While these associations are largely derived from observational studies and primarily reflect peripheral lipid measures, they raise the possibility that metabolic alterations in psychiatric populations may intersect with biological pathways relevant to brain function.

**Fig. 1:**
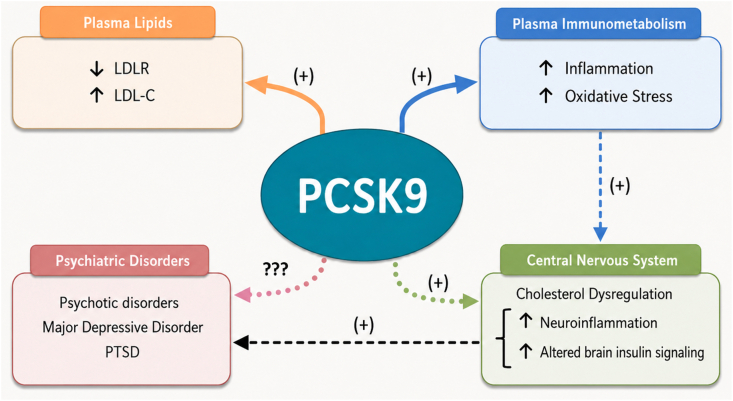
Schematic overview of PCSK9 at the intersection of lipid metabolism, immunometabolism, central nervous system processes, and psychiatric disorders. In the periphery, PCSK9 reduces LDLR availability and increases circulating LDL-C. PCSK9 is also associated with systemic immunometabolic alterations, including inflammation, oxidative stress, and insulin resistance. In the central nervous system, PCSK9-related pathways have been implicated in altered cholesterol homeostasis, neuroinflammatory signaling, and potential links with brain insulin resistance. These central processes may be relevant to psychiatric disorders; although the direct role of PCSK9 in psychiatric disease remains unresolved. Symbols: Solid arrows indicate established or relatively well-supported associations. Dashed arrows indicate indirect, context-dependent, or incompletely characterized relationships. Question marks indicate a hypothetical or unresolved direct link; + indicates stimulation. Abbreviations: PCSK9, proprotein convertase subtilisin/kexin type 9; LDLR, low-density lipoprotein receptor; LDL-C, low-density lipoprotein cholesterol; PTSD, post-traumatic stress disorder.

Against this background, the observation that olanzapine increases circulating PCSK9 levels provides a clinically salient entry point into a broader, largely unexplored question: whether PCSK9-related pathways may be relevant to psychiatric disorder biology more generally. The premise of this review is that PCSK9 may be most usefully conceptualized in psychiatry not primarily as an established causal mechanism, but as a framework for understanding how dyslipidemia, insulin resistance, low-grade inflammation, and central lipid-related signaling converge in psychiatric populations. In this narrative review, we therefore synthesize the existing clinical, experimental, and translational evidence to evaluate whether PCSK9 is best understood as a peripheral immunometabolic marker, a candidate mechanistic intermediary, or both, and to identify key gaps for future research. Fig. 1 provides a schematic overview of the conceptual framework guiding this review.

## 2 PCSK9 in peripheral and central lipid homeostasis

### 2.1 Peripheral PCSK9 and lipid homeostasis

PCSK9 is a serine protease best known for its central role in peripheral lipid metabolism. It is predominantly expressed in the liver, where it binds hepatic low-density lipoprotein receptors (LDLR) and targets them for lysosomal degradation, thereby reducing LDL clearance and increasing plasma LDL-C levels (Lambert et al., 2012). Genetic and clinical studies firmly establish this pathway as a major determinant of atherosclerotic cardiovascular disease risk: gain-of-function variants cause severe hypercholesterolemia, whereas loss-of-function variants confer lifelong reductions in LDL-C and marked cardiovascular protection (Hobbs et al., 2024).

### 2.2 Central PCSK9 and lipid homeostasis

Beyond its peripheral role, PCSK9 is also expressed at low levels within the CNS, including in neurons, astrocytes, oligodendrocytes, and microglia, where it exerts functions distinct from systemic lipoprotein regulation (Bell et al., 2023; O'Connell and Lohoff, 2020). The brain is largely isolated from circulating cholesterol by the blood–brain barrier and therefore relies on tightly regulated local cholesterol synthesis, transport, and recycling to maintain neuronal structure and function. Cholesterol is essential for myelination, neuronal membrane integrity, synapse formation, synaptic transmission, and long-term potentiation, and it also influences neurite outgrowth in a region-specific manner. Although peripheral cholesterol metabolism is driven by hepatic synthesis and dietary intake, communication between the brain and the periphery occurs through cholesterol metabolites which contribute to overall cholesterol homeostasis (Bell et al., 2023).

Within the CNS, cholesterol transport is mediated primarily by apolipoprotein E (ApoE)-containing lipoproteins and members of the low-density lipoprotein receptor family, including the low-density lipoprotein receptor (LDLR), low-density lipoprotein receptor-related protein 1 (LRP1), very-low-density lipoprotein receptor (VLDLR), and apolipoprotein E receptor 2 (ApoER2) (Mahley, 2016). PCSK9 has been shown to interact with these receptors, as well as with the fatty acid transporter CD36, suggesting a role in regulating neuronal cholesterol uptake and redistribution between neurons and glial cells. Although experimental findings are mixed regarding the extent to which PCSK9 directly induces degradation of these receptors in the brain, available evidence indicates that locally expressed PCSK9 can influence neuronal lipid availability, particularly during neurodevelopment and periods of synaptic remodeling (Jaafar et al., 2023).

Disruption of brain cholesterol homeostasis has profound consequences for synaptic plasticity and neurotransmission. Adequate cholesterol levels are required for dendritic spine stability, vesicle fusion, and efficient synaptic signaling, whereas cholesterol imbalance impairs long-term potentiation and synaptic growth (Pfrieger, 2003; Pfrieger and Ungerer, 2011). Altered PCSK9 activity may therefore compromise synaptic integrity and refinement, with downstream effects on cognition, emotional regulation, and behavior—domains frequently affected in psychiatric disorders such as schizophrenia, bipolar disorder, and MDD (Jaafar et al., 2023). Together, these observations provide the biological rationale for considering PCSK9 as a candidate link between lipid handling, synaptic biology, and psychiatric vulnerability.

## 3 PCSK9 in neuroinflammation and potential links with brain insulin resistance

### 3.1 Neuroinflammatory signaling

In addition to its effects on lipid handling, PCSK9 appears to play a role in neuroinflammatory processes that is partly independent of its canonical function in cholesterol metabolism. Experimental studies demonstrate that PCSK9 can promote innate immune activation via TLR4–NF-κB–dependent pathways, leading to increased production of pro-inflammatory cytokines and enhanced microglial and astrocytic activation (Ricci et al., 2018; Scalise et al., 2021; Vilella et al., 2024). In animal models of Alzheimer's disease and cerebral ischemia, genetic deletion or inhibition of PCSK9 reduces neuroinflammation and amyloid burden, with relatively modest effects on total brain cholesterol content (Luo et al., 2025; Vilella et al., 2024; Y. Zheng et al., 2024).

### 3.2 Potential links with brain insulin resistance

Emerging evidence also suggests that PCSK9 may intersect with pathways relevant to brain insulin resistance, a feature increasingly recognized in neurodegenerative and affective disorders (Mansur et al., 2021; McIntyre, 2021). Among the LDLR-family receptors involved in central lipid homeostasis, LRP1 also plays a central role in neuronal insulin and insulin-like growth factor signaling, thereby linking cholesterol trafficking to synaptic energy metabolism and plasticity (Au et al., 2017). Under conditions of increased PCSK9 activity, PCSK9 could plausibly reduce the availability of these receptors, potentially exacerbating synaptic vulnerability, impaired energy metabolism, and altered plasticity associated with brain insulin resistance (O'Connell and Lohoff, 2020). These effects may be particularly relevant in APOE4 carriers, in whom elevated cerebrospinal fluid (CSF) PCSK9 levels have been linked to altered oxysterol and lipid profiles in Alzheimer's disease (Papotti et al., 2024). Although direct mechanistic evidence in humans remains limited, these observations support the possibility that central PCSK9 regulation may be altered under pathological conditions relevant to impaired brain insulin signaling.

## 4 Peripheral PCSK9 and dyslipidemia in psychiatric disorders

Limited evidence suggests that circulating PCSK9 reflects broader metabolic and inflammatory states relevant to psychiatric disorders. From a psychiatric perspective, an important question is whether PCSK9-related pathways may provide a mechanistic or biomarker framework linking lipid abnormalities to psychiatric symptomatology across diagnostic groups.

### 4.1 Major depressive disorder

Direct evidence linking PCSK9 to MDD remains limited. Observational work has shown that higher plasma PCSK9 levels are associated with greater depressive symptom severity and insulin resistance, particularly in individuals with obesity or prediabetes (Macchi et al., 2020). Additional studies have reported elevated PCSK9 concentrations in patients with MDD together with increased oxidative stress and reduced antioxidant capacity (Habibitabar et al., 2024). By contrast, Mendelian randomization analyses modeling the on-target effects of PCSK9 inhibition have not supported a causal relationship between PCSK9 and mood disorders, suggesting that circulating PCSK9 is more likely to reflect a broader immunometabolic phenotype associated with depressive symptoms than to represent a primary causal driver of depression (Aman et al., 2024).

This interpretation is supported by the metabolic abnormalities consistently observed in MDD. Among mood disorders, MDD has been most consistently linked to metabolic abnormalities that overlap with known correlates of elevated circulating PCSK9. Meta-analytic evidence indicates that serum lipid alterations in MDD are heterogeneous, with inconsistent associations for total cholesterol and LDL-C, but more reproducible elevations in triglycerides, particularly in metabolically burdened or psychotropic-exposed populations (Bharti et al., 2021; Persons and Fiedorowicz, 2016; Shin et al., 2008; Xu et al., 2024). These patterns provide a biologically plausible context for PCSK9 involvement.

### 4.2 Psychotic disorders and antipsychotic exposure

Direct psychiatric evidence involving PCSK9 comes mainly from antipsychotic treatment studies, particularly those examining olanzapine-induced metabolic effects, rather than from psychotic disorders themselves. Olanzapine, in particular, has been associated with increased circulating PCSK9 levels in patients with schizophrenia, together with dyslipidemia and hepatic lipid dysregulation (J. Huang et al., 2022; P. Huang et al., 2024; Zhu et al., 2022). These observations are clinically important because they suggest that PCSK9 may be engaged by treatments that are themselves strongly linked to metabolic toxicity.

This is particularly relevant in psychotic disorders, where PCSK9 intersects both with disease-related metabolic abnormalities and with antipsychotic-induced dysmetabolism. In antipsychotic-naïve individuals with first-episode psychosis, lower total and LDL cholesterol levels have been reported alongside elevated triglyceride concentrations (Pillinger et al., 2017). In chronic schizophrenia, meta-analytic evidence indicates markedly elevated triglyceride and remnant cholesterol levels accompanied by systemic inflammation; however, lipid abnormalities and inflammatory markers seem to contribute independently to cardiometabolic risk (Wang et al., 2024). This makes psychotic disorders, particularly in the context of SGA exposure, a clinically relevant setting in which to study PCSK9 as a marker of treatment-related metabolic burden.

### 4.3 Post-traumatic stress disorder

In post-traumatic stress disorder (PTSD), direct data on circulating PCSK9 are currently lacking, but the disorder is characterized by a metabolic profile that is compatible with PCSK9-related dysregulation. Meta-analyses consistently describe elevated total cholesterol, LDL-C, and triglycerides, together with reduced HDL-C, relative to healthy controls (Bharti et al., 2022). Although these findings do not establish a role for PCSK9, they place PTSD within a group of psychiatric conditions marked by adverse peripheral lipid metabolism and inflammatory burden, both of which are biologically linked to circulating PCSK9 in non-psychiatric populations.

Taken together, the available literature suggests that peripheral PCSK9 is better understood as a marker of systemic immunometabolic burden than as a disorder-specific causal factor. Although direct evidence for altered peripheral PCSK9 remains limited, it may be most relevant in psychiatric subgroups characterized by prominent metabolic dysfunction.

## 5 Central PCSK9 and neuropsychiatric relevance

Whereas circulating PCSK9 is primarily relevant as a peripheral immunometabolic marker, the potential role of PCSK9 within the CNS remains less clear. PCSK9 is expressed at low levels in the brain under physiological conditions and appears to be regulated independently from the circulation, with low and relatively stable CSF concentrations compared with the marked diurnal variation observed in plasma (Y. Q. Chen et al., 2014; Jaafar et al., 2023). This compartmentalization is important, because it implies that any contribution of PCSK9 to psychiatric disorder may involve distinct central and peripheral processes rather than a single unified pathway.

### 5.1 Central PCSK9 in preclinical models

Preclinical data provide the strongest support for a functionally relevant role of PCSK9 in the brain. In models of cerebral ischemia and Alzheimer's disease, genetic deletion or pharmacological inhibition of PCSK9 reduces neuroinflammation, attenuates pathological burden, and improves functional outcomes, often without major changes in total brain cholesterol content (Apaijai et al., 2019; Luo et al., 2025; Vilella et al., 2024; Zheng et al., 2024). Behavioral and synaptic studies further support this view. Global PCSK9 knockout mice show sex-dependent anxiety- and depression-like phenotypes, while neuron-specific PCSK9 deletion has been associated with impaired cognition and altered ApoER2 synaptic localization (Nasini et al., 2026; Pelucchi et al., 2025). Although these models are not models of psychiatric disorders in the strict sense, they indicate that central PCSK9 can influence behavioral domains relevant to affective and cognitive dysfunction.

### 5.2 CNS lipid abnormalities in psychiatric disorders

Although direct evidence for altered central PCSK9 in psychiatric disorders is lacking, human studies suggest disruption of related central lipid and cholesterol pathways. Post-mortem studies in schizophrenia and bipolar disorder have shown altered membrane lipid composition across multiple brain regions, including prefrontal changes in free fatty acids and phosphatidylcholine, increased white-matter ceramides, and thalamic reductions in phosphatidylcholine, sphingomyelin, and galactocerebrosides with increased phosphatidylserine, consistent with impaired membrane integrity, myelination, and oligodendrocyte function (Schmitt et al., 2004; Schwarz et al., 2008).

Disturbances in brain–periphery cholesterol turnover have also been implicated by studies of oxysterols, blood–brain barrier–permeable cholesterol metabolites that regulate central cholesterol homeostasis (Delac and Maioli, 2025). Elevated 24S-hydroxycholesterol, reduced 27-hydroxycholesterol, and a higher 24S/27-hydroxycholesterol ratio have been reported in schizophrenia and in individuals at clinical high risk for psychosis, with the ratio correlating with symptom severity (Sun et al., 2021). Altered oxysterol profiles have also been linked to affective symptoms across diagnostic boundaries, including associations with depressive symptoms in Parkinson's disease and with disease state, treatment response, and clinical improvement in MDD (Griffiths et al., 2021; Sun et al., 2023).

### 5.3 Central PCSK9 in related brain disorders

Human studies directly examining central PCSK9 are limited. In alcohol use disorder, CSF PCSK9 levels are elevated and show a modest correlation with plasma concentrations, suggesting that partial peripheral-central coupling may occur under pathological conditions (Lee et al., 2019). In Alzheimer's disease, CSF PCSK9 has been associated with lipoprotein measures, APOE4 status, and Alzheimer-related biomarkers, including at preclinical stages (Papotti et al., 2024; Picard et al., 2019). These observations do not establish relevance for psychiatric disorders directly, but they do support the concept that central PCSK9 becomes dysregulated in brain disorders marked by inflammation, altered energetics, or lipid disturbance. Peripheral and central PCSK9 systems appear mechanistically distinct and are not tightly coupled under physiological conditions (Jaafar et al., 2023), but coupling may emerge in disease through shared inflammatory regulation, altered barrier function, or regulated exchange at the choroid plexus rather than passive diffusion across an intact blood–brain barrier (Solár et al., 2020). Overall, central PCSK9 should be viewed not as an established mechanism in psychiatric disorders, but as a biologically plausible translational hypothesis that warrants direct investigation across plasma, CSF, and, where possible, brain tissue.

## 6 Lipid-lowering therapy in psychiatric disorders

Collectively, psychiatric disorders have been linked to dysregulated lipid metabolism, including peripheral lipid abnormalities and PCSK9-related pathways. This raises the question of whether lipid-lowering therapies could be relevant in the context of psychiatric disorders.

### 6.1 Statins

Statins have been extensively studied in relation to psychiatric outcomes. Meta-analyses of randomized controlled trials indicate that statin monotherapy does not improve depressive symptoms (De Giorgi et al., 2022). When used adjunctively with antidepressants, modest benefits on depressive symptoms have been reported (Xiao et al., 2023).

In schizophrenia, adjunctive statin therapy has shown mixed results. While some meta-analyses report improvements in negative and total symptom scores (Peng et al., 2024; Shen et al., 2018), others—particularly those focusing on simvastatin—find no significant benefit on core psychopathology (J. Chen et al., 2024). A possible explanation for these discrepancies in effect may be patient heterogeneity, including the absence of an immunometabolic profile that would confer responsiveness to statin therapy (van Bruggen and McIntyre, 2026). Also, statins are known to increase plasma PCSK9 levels (Sahebkar et al., 2015). Given that simvastatin is lipophilic and can cross the blood–brain barrier, it is conceivable that central PCSK9 upregulation could offset other immunomodulatory effects of statins, although this hypothesis remains speculative.

### 6.2 PCSK9 inhibitors

PCSK9 inhibitors act primarily in the peripheral circulation and do not appreciably penetrate the CNS under physiological conditions. Evidence regarding their neuropsychiatric effects is limited and mixed. Pharmacovigilance studies report neuropsychiatric adverse events (di Mauro et al., 2021; Gouverneur et al., 2023) whereas randomized trial meta-analyses do not indicate increased neurocognitive risk (van Bruggen et al., 2020). Overall, current evidence does not support a major direct impact of PCSK9 inhibitors on psychiatric outcomes.

## 7 Discussion and conclusions

This narrative review brings together experimental, genetic, and clinical evidence suggesting that PCSK9-related pathways may be relevant to the biology of psychiatric disorders, particularly in the context of metabolic and inflammatory dysregulation. Taken together, and as summarized in Fig. 1, the available evidence most strongly supports a role for peripheral PCSK9 as a marker of systemic immunometabolic burden, whereas evidence for a direct central role in psychiatric disorders remains limited.

A central unresolved issue concerns compartmentalization. Circulating PCSK9 does not cross an intact blood–brain barrier, and CSF concentrations are low and independently regulated from plasma levels. However, blood–brain barrier dysfunction has been reported in subsets of patients with severe psychiatric illness, particularly in the context of inflammation, metabolic disease, substance use, or vascular comorbidity (Zhao et al., 2022). Under such conditions, peripheral PCSK9-related lipid or inflammatory signals could plausibly exert indirect or regulated central effects. Whether and to what extent such peripheral–central interactions occur in psychiatric populations remains uncertain, and the absence of studies simultaneously assessing PCSK9 across plasma, CSF, and brain tissue therefore constitutes a critical knowledge gap rather than evidence against central relevance.

The available evidence also has important translational implications. A particularly intriguing finding is provided by glucagon-like peptide-1 (GLP-1) receptor agonists. Liraglutide has been shown to reduce circulating PCSK9 concentrations in patients with diabetes (Vergès et al., 2022), and GLP-1 receptor agonists are known to exert central effects. These observations introduce the testable hypothesis that GLP-1–mediated metabolic modulation may influence PCSK9-related pathways relevant to brain inflammation or energetics, although direct evidence for such effects is currently lacking.

In conclusion, current evidence supports a model in which plasma PCSK9 reflects immunometabolic dysregulation relevant to psychiatric vulnerability rather than acting as a direct neuropsychiatric effector. There is currently no direct evidence for a causal role of PCSK9 within the CNS in psychiatric disorders. Instead, PCSK9 is best viewed as a peripheral biomarker situated at the interface of metabolic and inflammatory processes. Further studies are warranted to better characterize PCSK9-related processes within the central compartment in the context of psychiatric disorders.

## Funding

This research did not receive any specific grant from funding agencies in the public, commercial, or not-for-profit sectors.

## CRediT authorship contribution statement

**Folkert H. van Bruggen:** Conceptualization, Writing – original draft, Writing – review & editing. **Roger S. McIntyre:** Conceptualization, Supervision, Writing – review & editing.

## Declaration of competing interest

Folkert van Bruggen: none.

Roger McIntyre has received research grant support from CIHR/GACD/National Natural Science Foundation of China (NSFC) and the Milken Institute; speaker/consultation fees from Lundbeck, Janssen, Alkermes, Neumora Therapeutics, Boehringer Ingelheim, Sage, Biogen, Mitsubishi Tanabe, Purdue, Pfizer, Otsuka, Takeda, Neurocrine, Neurawell, Sunovion, Bausch Health, Axsome, Novo Nordisk, Kris, Sanofi, Eisai, Intra-Cellular, NewBridge Pharmaceuticals, Viatris, Abbvie and Atai Life Sciences**.**

## Data Availability

No data was used for the research described in the article.
